# Impact of Short‐Chain Fatty Acids on Glucose, Fatty Acid and Leucine Metabolism in Primary Human Myotubes

**DOI:** 10.1002/edm2.70042

**Published:** 2025-03-07

**Authors:** Ragna Husby Tingstad, Oliwia Witczak, Siver Beajani, Seung Hee Seo, Nils Gunnar Løvsletten, Christine Skagen, Mari Charlotte Wik Myhrstad, Vibeke Helen Telle‐Hansen, Lars Eide, Arild Christian Rustan, Vigdis Aas

**Affiliations:** ^1^ Department of Life Sciences and Health, Faculty of Health Sciences Oslo Metropolitan University Oslo Norway; ^2^ Section for Pharmacology and Pharmaceutical Biosciences, Department of Pharmacy University of Oslo Oslo Norway; ^3^ Department of Nursing and Health Promotion, Faculty of Health Sciences Oslo Metropolitan University Oslo Norway; ^4^ Department of Medical Biochemistry, Institute of Clinical Medicine, Faculty of Medicine University of Oslo Oslo Norway

**Keywords:** acetate, butyrate, energy metabolism, leucine, myotubes, propionate, protein synthesis, short‐chain fatty acids

## Abstract

**Introduction:**

Short‐chain fatty acids (SCFAs) are small molecule metabolites mainly produced during microbial fermentation of dietary fibre in the gut and have been shown to have a beneficial impact on human health. The aim of this study was to evaluate the effect of SCFAs on human skeletal muscle energy metabolism.

**Methods:**

Primary human myotubes were analysed for glucose and fatty acid (oleic acid) metabolism, as well as insulin sensitivity and protein synthesis in the presence or absence of SCFAs.

**Results:**

The most pronounced effects of SCFAs were observed on ^14^C‐oleic acid uptake and oxidation, as well as ^14^C‐leucine uptake and protein synthesis, following butyrate treatment. Butyrate increased ^14^C‐leucine accumulation twofold, potentially due to protein incorporation. On the other hand, the conversion of ^14^C‐leucine into free fatty acids was reduced by more than 50% by butyrate. Both ^14^C‐acetate and ^14^C‐butyrate were shown to be taken up and utilised by primary human myotubes. None of the SCFAs were found to influence glucose metabolism or insulin effects.

**Conclusion:**

The results from the current study thus suggest that among the SCFAs, butyrate emerges as the most powerful SCFA in regulating primary human myotube metabolism.

## Graphical abstract

SCFAs can be metabolised to a different extent, and butyrate emerges as the most effective SCFA in affecting both oleic acid and leucine metabolisms in cultured human skeletal muscle cells.

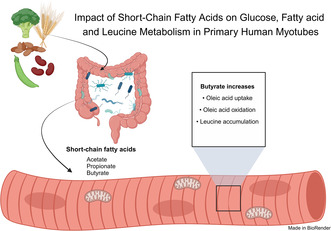

## 1 Introduction

Dietary fibre ingestion has several beneficial effects on human health [1], such as improving gut function, reducing the risk of a range of diseases, and reducing blood glucose following food intake [2]. Many dietary fibres, also called prebiotics, are non‐digestible by human gut epithelial cells, but support the promotion of favourable bacterial species in the gut [3, 4] and are fermented by gut microorganisms to short‐chain fatty acids (SCFAs).

SCFAs are carboxylic acids with less than five carbon atoms [5]. The three major SCFAs that are produced are acetate, propionate and butyrate, accounting for > 90% of the total colonic SCFA content [6]. Following the intestinal release of SCFAs into the portal vein [7], acetate is the most abundant, with concentrations in blood ranging from 170 to 260 μmol/L, whereas propionate and butyrate measure 4–30 μmol/L [7, 8]. As with fibre, SCFAs have been found to elicit several beneficial processes in the human body. These include enhancing intestinal barrier function [9, 10], and lowering the risk of cardiovascular disease [11], colonic cancer [12], and type 2 diabetes (T2D) [13].

The monocarboxylate transporters (MCTs) MCT1, MCT2 and MCT4 have been found to transport SCFAs into cells [14, 15], and are all expressed as proteins in skeletal muscle [16]. This suggests that SCFAs not only function as energy sources for colonocytes, but might also be utilised systemically, such as in skeletal muscle tissue [17]. By this means, the gut microbiota can affect the amount of energy extracted from the food and enhance energy supply. In addition, several studies have investigated the effects of SCFAs on skeletal muscle mass and phenotype [18, 19, 20, 21]. Previous in vivo studies have shown that antibiotics reduce muscle mass in mice [22], indicating the involvement of the microbiota or their products in maintaining muscle mass. SCFAs can increase muscle mass in germ‐free mice [22], or prevent skeletal muscle atrophy in ageing mice [23]. In addition, diet supplementation with both butyrate and acetate induced an oxidative phenotype [18, 19, 20], as suggested by an increased expression of type 1 fibres and upregulation of genes involved in fatty acid β‐oxidation [18, 20] in mice.

In addition to functioning as energy substrates themselves, SCFAs have also a regulatory effect on energy metabolism [18, 24]. Peroxisome proliferator‐activated receptor γ (*PPARγ*) mRNA and protein expression was increased by SCFAs in C2C12 cells [24], while in liver and adipose tissue, SCFAs downregulated *PPARγ*, resulting in increased lipid oxidation, energy expenditure, and regulation of lipogenesis [24]. Both *PPARδ* mRNA and its co‐activator peroxisome proliferator‐activated receptor‐γ coactivator‐1α (*PGC‐1α*) protein expression were found to be increased following butyrate treatment of the rat myotube cell line L6. Butyrate also enhanced mitochondrial lipid oxidation in mice fed a high fat diet [18]. As previously reviewed [23], SCFAs have been shown to increase both insulin‐stimulated and insulin‐independent glucose uptake in L6 and C2C12 (from mice) cell lines. In addition, acetate treatment increased *GLUT4* gene and protein expression in L6 myotubes [25].

SCFAs can also signal through G protein‐coupled receptors (GPR), like GPR41 and GPR43 [26, 27]. GPR41 has a differential affinity for SCFAs (propionate > butyrate > acetate) [28], whereas GPR43 shows equal affinity for the SCFAs [28]. Although the mRNA of the receptors has been found to be expressed in skeletal muscle tissues, the protein expression has not been demonstrated [27, 29]. The receptors appear to be differentially expressed in various tissues [30], but their role in skeletal muscle metabolism remains elusive [23]. Notably, GPR41‐deficient male mice have an increase in body fat with reduced glucose tolerance [31], while mice lacking GPR43 become obese when fed a normal diet, and mice over‐expressing GPR43 remain lean despite being fed a high‐fat diet [32]. This suggests a role for GPR43 in energy homeostasis.

As skeletal muscle is a large organ for storage and utilisation of energy, both basally and under the regulation of insulin, its possible influence by SCFAs is of interest. The aim of the present study was to determine if the SCFAs acetate, propionate, and butyrate might have a direct impact on the energy metabolism of glucose, oleic acid and leucine, as well as insulin sensitivity and protein synthesis in primary human myotubes isolated from healthy donors.

## 2 Materials and Methods

### 2.1 Materials

Dulbecco's modified eagle medium (DMEM)‐glutamax low glucose, Dulbecco's phosphate buffered saline (DPBS) (with CaCl_2_ and MgCl_2_), amphotericin B, Applied Biosystem's High‐Capacity cDNA reverse transcription kit, foetal bovine serum (FBS), hydroxyethyl piperazineethanesulfonic acid (HEPES), Pierce BCA Protein Assay kit, Human EGF Recombinant Protein, Dulbecco's modified eagle medium (DMEM)‐no glucose and real‐time qPCR primers were from ThermoFisher Scientific (ThermoFisher Scientific, Foster City, CA, US). Penicillin–streptomycin, DL‐β‐hydroxybutyric acid sodium salt, 96‐well and 6‐well Corning CellBIND plates, gentamicin, NaOH, oleic acid, glucose, bovine serum albumin (BSA), L‐carnitine, dexamethasone, and glycogen from rabbit liver were from Sigma‐Aldrich (St. Louis, MO, US). Insulin (100 IE/mL) was from Novo Nordisk (Bagsvaerd, Denmark). RNeasy Mini Kit and QuantiNova SYBR Green RT‐PCR kit were from Qiagen (Düsseldorf, Germany). Bio‐Rad Protein Assay Dye Reagent Concentrate, BioRad Criterion TGX precast gels, BioRad precision plus protein dual colour and Chemidoc XRS+ Imager were from BioRad (Hercules, CA, US). PerkinElmer 2450 MicroBeta^2^ scintillation counter, Unifilter‐96 GF/B, 96‐well Isoplate, ScintiPlate‐96 TC, Ultima Gold LSC scintillation liquid, OptiPhase Supermix and TopSeal‐A transparent film were from PerkinElmer (Shelton, CT, US). Primary antibodies against p‐Akt (Ser473) (#9271) and Akt (#9272) and the HRP‐conjugated secondary antibody (#7074) were from Cell Signalling Technology (Danvers, MA, US). The primary antibody against α‐tubulin (#GR3194994‐1) was from Abcam (Cambridge, UK). Image J was from the National Institutes of Health (Bethesda, MD, US).

### 2.2 Primary Human Skeletal Muscle Cell Cultures

Human satellite cells were isolated from *M. vastus lateralis* from biopsies of healthy donors, as described previously by Gaster et al. [33]. Human skeletal muscle biopsies were obtained after informed written consent from the patient and approval by the Regional Committee for Medical and Health Research Ethics South‐East, Oslo, Norway (reference number: REK11959). The study was conducted in accordance with the guidelines of the Declaration of Helsinki. The 5 donors (60% female) were 48 ± 4 (mean ± SEM) years of age, with a body mass index of 27 ± 2 kg/m^2^ and had a fasting plasma glucose of 4.0 ± 0.2 mmol/L. For the experiments assessing uptake and oxidation of acetate, the muscle cells were obtained from 6 male donors aged 53 ± 1.8 years with a body mass index of 23 ± 0.7 kg/m^2^ and had a fasting plasma glucose of 5.2 ± 0.1 mmol/L (ethical approval from the West of Scotland Research Ethics Committee 4 (reference number: 16/WS/0002)). The myoblasts were proliferated until ~80% confluence, in 96‐well or 6‐well CellBind microplates, as described previously [34], or in a slightly modified proliferation medium containing DMEM‐Glutamax low glucose supplemented with HEPES (20 mM), 10% foetal bovine serum, human epidermal growth factor (10 ng/mL), dexamethasone (0.39 μg/mL), bovine serum albumin (0.05%), gentamicin (50 μg/mL), penicillin/streptomycin (50 units/mL) and amphotericin B (1.25 μg/mL). Cell differentiation from myoblasts to myotubes was initiated as described previously [34]. Experiments were carried out on day 7 of differentiation. For all experimental set‐ups, cells from each donor served as controls. These control cells were incubated under the same conditions as the SCFA‐treated cells but received only differentiation medium without SCFAs.

### 2.3 Substrate Uptake and Oxidation

To assess the utilisation of energy sources by human skeletal muscle, substrate oxidation measurements were carried out in differentiated myotubes as described previously [35]. For assessing the SCFAs effect on glucose and oleic acid uptake and oxidation, the myotubes were treated with 100 μM of either acetate, propionate, or butyrate for 24 h before the substrate oxidation assay. Either [1‐^14^C]acetate (2 μCi/mL, 100 μM), [1‐^14^C]butyrate (0.6 μCi/mL, 12 μM), D‐[U‐^14^C]glucose (1 μCi/mL, 200 μM) or [1‐^14^C]oleic acid (1 μCi/mL, 100 μM) were added to DPBS with 20 mM HEPES. The cells were washed with 37°C DPBS before a 96‐well Unifilter microplate soaked with 1 M NaOH (20 μL/well) was fixed on top of the cell plate, and the cells were then incubated in a humidified 5% CO_2_ atmosphere at 37°C for 4 h. The radio‐labelled medium was then removed, and the cells were washed twice in PBS at room temperature before being lysed in 0.1 M NaOH and frozen at −20°C. Both the Unifilter containing the trapped CO_2_ and the cell lysates were counted by liquid scintillation using a PerkinElmer 2450 MicroBeta^2^ counter. Protein concentrations were measured with the BioRad protein assay as described previously [36] using a PerkinElmer VICTOR^3^ 1420 Multilabel counter. The substrate uptake (calculated as the sum of CO_2_ and any remaining cell associated substrate) and oxidation were presented/given as nmol/mg protein.

### 2.4 Glycogen Synthesis

The myotubes were differentiated in 6‐well CellBind culture plates, as described above, and after 6 days of differentiation, the cells were treated with 100 μM of either acetate, propionate, or butyrate for 24 h. The myotubes were then incubated for 1 h in serum‐free DMEM‐Glutamax prior to the addition of D‐[^14^C(U)]glucose (1.0 μCi/mL) for 2 h with or without 100 nM insulin. The cells were washed twice with PBS and lysed in 0.5 mL KOH (1 M), and the glycogen precipitation measurement was carried out as described previously [37]. Each sample (250 μL) was added to 3 mL Ultima Gold LSC scintillation solution, and the radioactivity was counted by a Packard TriCarb 1900 TR Liquid Scintillation Analyser. The protein content was measured using the Pierce BCA Protein Assay kit according to the manufacturer's protocol.

### 2.5 Western Blotting

For Western blotting, the myotubes were proliferated and differentiated in 6‐well CellBind culture plates, as described above, in the presence or absence of 100 μM of either acetate, propionate, or butyrate for 24 h. The cells were then incubated for 15 min in DMEM‐Glutamax in the presence or absence of 100 nM insulin, before the samples were harvested in ice‐cold radioimmunoprecipitation assay (RIPA) buffer supplemented with protease inhibitors (Roche complete protease inhibitor cocktail tablets) and phosphatase inhibitors (final concentration of 50 mM NaF, 10 mM NaPPi and 1 mM Na_3_Vo_4_). Protein concentrations were measured using the Pierce BCA protein assay kit before 4X SDS loading dye was added to the samples. Cell protein (15 μg) from each sample was loaded onto 4%–20% BioRad Criterion TGX precast gels, using the BioRad precision plus protein dual colour as a protein standard. The samples were transferred from the gels onto polyvinylidene fluoride (PVDF) membranes, and the membranes were incubated with primary antibodies against P‐Akt (Ser473) (1:1000), Akt (1:1000) and α‐tubulin (1:1000) at 4°C overnight. An HRP‐conjugated secondary antibody (1:2000) was added to the membranes following washing, and the antibody reactive bands were detected with chemiluminescence from the Bio‐Rad ImmunStar WesternC kit using the Bio‐Rad Chemidoc XRS+ system, prior to analysis using Image J (version 1.53t) software.

### 2.6 Protein Synthesis

Protein synthesis was measured using both scintillation proximity assay (SPA) and protein precipitation. SPA was carried out to measure real‐time accumulation of radiolabelled leucine (^14^C) by adherent cells. Radioactivity concentrated closer to the scintillator embedded in the plastic bottom of each well provides a stronger signal than the radiolabelled substrate in the culture medium, as described previously [35]. Myotubes were cultured on 96‐well ScintiPlate tissue culture plates, and SPA was used to measure the cellular accumulation of leucine. The cells were treated with 100 μM of either acetate, propionate, or butyrate for 24 h prior to the experiment. On day 7 of differentiation, the cells were given [^14^C]leucine (1 μCi/mL, 0.8 mM) in the presence or absence of the SCFAs. A concentration of 0.8 mM leucine was chosen to align with the leucine concentration in the DMEM‐Glutamax used. The time course for [^14^C]leucine accumulation was measured by a PerkinElmer 2450 MicroBeta^2^ scintillation counter, and the plate was counted at 0, 2, 4 and 6 h. The amount of protein per well was determined according to Bradford [36], and the amount of accumulated ^14^C leucine was normalised to total cell protein content.

To measure ^14^C‐leucine incorporation into cellular proteins, myotubes were differentiated in 24‐well CellBind culture plates, as described above, and on day 6 of differentiation, the cells were treated with 100 μM of either acetate or butyrate or 200 μM propionate, for 24 h together with [^14^C]leucine (1 μCi/mL, 0.8 mM). The cells were lysed in 0.01% SDS, and the total cell protein content was measured using the Pierce BCA protein assay kit. Cellular proteins were precipitated using 6% BSA and 50% trichloroacetic acid, and the protein associated with [^14^C]leucine was measured by liquid scintillation using a Packard Tri‐Carb 1900 TR (Perkin Elmer). The [^14^C]leucine protein was normalised against the total cell protein content.

### 2.7 Lipid Distribution

Myotubes were cultured on 6‐well CellBind culture plates and treated with 100 μM butyrate for 24 h together with [^14^C]leucine (1 μCi/mL, 0.8 mM). The cells were washed twice in cold PBS before they were harvested in 0.1% SDS, and the total cell protein concentration was measured using the Pierce BCA Protein Assay kit according to the manufacturer's protocol. The remaining cell lysate was mixed with a 2:1 solution of chloroform: methanol and 0.6% foetal calf serum (FCS) for the extraction of lipids. The lipids were separated by thin‐layer chromatography (TLC), as described previously [38], from the homogenised cell fractions using free fatty acids, cholesterol ester, mono‐, di‐, and triglycerides as standards. The lipid‐associated [^14^C]leucine was measured by liquid scintillation using a Packard Tri‐Carb 1900 TR (Perkin Elmer), and the samples were normalised against the total cell protein content.

### 2.8 mRNA Quantification by Real Time qPCR

Human satellite cells were proliferated and differentiated in 6‐well CellBind culture plates, as described above, and incubated for 24 h with 100 μM of either acetate, propionate, or butyrate. Total RNA was extracted using the Qiagen RNeasy mini kit, following the manufacturer's protocol. The RNA was reversely transcribed using the Applied Biosystem's High‐Capacity cDNA reverse transcription kit, following the manufacturer's protocol and using an Applied Biosystems 2720 Thermal Cycler. Real‐time qPCR was carried out using both the Qiagen's QuantiNova SYBR Green RT‐PCR kit and the Applied Biosystems StepOnePlus Real‐time PCR system with Power SYBR Green PCR Master Mix, as described in the manufacturer's protocols, using a Stratagene MX3000p qPCR cycler. *GAPDH* or *RPLP0* was analysed as a housekeeping gene and used for normalising the expression levels of *PDK4*, *CPT1B, PPARD, PPARG, CD36, GLUT1* and *GLUT4*. The following human primers were used (see Table 1).

**TABLE 1:** Primer sequences for gene expression analysis by Real‐time PCR.

Gene name (gene symbol)	Accession number	Forward primer (5′‐3′)	Reverse primer (5′‐3′)
Pyruvate dehydrogenase kinase 4 (*PDK4*)	BC040239	TTT CCA GAC CAA CCA ATT CAC A	TGC CCG CAT TGC ATT CTT A
Carnitine palmitoyltransferase 1B (*CPT1B*)	NM004377	CAA AAT TCC CTT CCT GCT CCA AC	CGC TTT GGA AAC CAC ATC CG
Peroxisome proliferator‐activated receptor δ (*PPARD*)	BC002715	AGC ATC CTC ACC GGC AAA	ATG TCT CGA TGT CGT GGA TCA C
Peroxisome proliferator‐activated receptor γ (*PPARG*)	L40904	AGC CTG CGA AAG CCT TTT G	ATT CCA GTG CAT TGA ACT TCA CA
Platelet glycoprotein 4 (*CD36*)	L06850	AGT CAC TGC GAC ATG ATT AAT GGT	CTG CAA TAC CTG GCT TTT CTC AA
Solute carrier family 2, facilitated glucose transporter member 1 (*GLUT1* or *SLC2A1*)	K03195	CAG CAG CCC TAA GGA TCT CTC A	CCG GCT CGG CTG ACA TC
Solute carrier family 2, facilitated glucose transporter member 4 (*GLUT4* or *SLC2A4*)	M20747	ACC CTG GTC CTT GCT GTG TT	ACC CCA ATG TTG TAC CCA AAC T
Glyceraldehyde‐3‐phosphate dehydrogenase (*GAPDH*)	NM002046	TGC ACC ACC AAC TGC TTA GC	GGC ATG GAC TGT GGT CAT GAG
Acidic ribosomal phosphoprotein P0 (*RPLP0*)	M17885	CCA TTC TAT CAT CAA CGG GTA CAA	AGC AAG TGG GAA GGT GTA ATC C

### 2.9 Statistical Analysis

Comparisons between SCFA‐treated myotubes and controls were performed by either one‐sample or paired Student's *t*‐tests. The level of significance was set to *α* = 0.05, and *p* < 0.05 was considered statistically significant. Data in figures are presented as mean ± SEM unless stated otherwise, and the number of individual experiments and technical replicates for the experiments are listed in the figure legends. Graphs were prepared using GraphPad Prism 8.3.0 for Windows (GraphPad Software Inc., San Diego, CA, US).

## 3 Results

### 3.1 Effects of SCFAs on Glucose Metabolism

**FIGURE 1:**
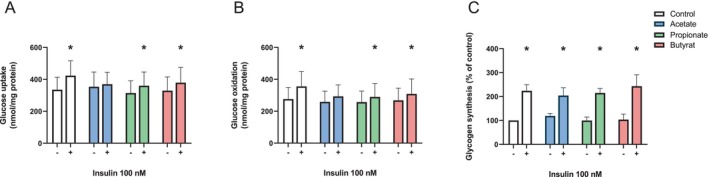
Glucose metabolism in cultured human myotubes following short‐chain fatty acid (SCFA) treatment in the presence or absence of insulin (100 nM). Human myotubes were cultured in 6‐well CellBind culture plates and treated with 100 μM of either acetate, propionate, or butyrate for 24 h before the uptake and oxidation assays. The myotubes were then incubated with D‐[^14^C(U)]glucose (1 μCi/mL, 200 μM) in the presence or absence of 100 nM insulin for 4 h. Glucose uptake (A) was assessed as the sum of both oxidised D‐[^14^C(U)]glucose and the remaining cell‐associated radioactivity; data are given as mean ± SEM. Glucose oxidation (B) refers to the oxidised D‐[^14^C(U)]glucose trapped in a filter as CO_2_ and counted by liquid scintillation; data are given as mean ± SEM. Glycogen synthesis (C) was assessed in 6‐well plates and refers to precipitated glycogen containing D‐[^14^C(U)]glucose measured by liquid scintillation and related to unstimulated control myotubes (% of control). The average myotube glycogen content in the unstimulated control myotubes was 4.4 ± 1.3 nmol/mg cell protein. *n* = 3–5 individual experiments with 3–4 technical replicates per experiment. *p < 0.05 vs. basal, by paired Student's *t*‐test.

To evaluate SCFAs' impact on glucose metabolism in human myotubes, the differentiated muscle cells were treated with 100 μM of either acetate, propionate, or butyrate for 24 h prior to incubation with D‐[^14^C(U)]glucose in the presence or absence of 100 nM insulin for 4 h (Figure 1A,B). The selected SCFA concentrations and duration of treatment were based on time course and concentration experiments (Figures S1 and S2). None of the SCFAs influenced basal glucose uptake (Figure 1A) or glucose oxidation in myotubes (Figure 1B). However, insulin‐stimulated glucose uptake and oxidation were not apparent after exposure to acetate (Figure 1A,B). On the other hand, in both control and myotubes exposed to propionate and butyrate, insulin increased glucose uptake and oxidation to the same extent (15%–20%). Basal and insulin‐stimulated glycogen synthesis were also determined in control myotubes and in myotubes treated with SCFAs (Figure 1C). As expected, insulin increased glycogen synthesis about 2‐fold, and both baseline and insulin‐stimulated glycogen synthesis appeared to be unchanged by treatment with SCFAs (Figure 1C).

### 3.2 Effects of SCFAs on Insulin Signalling

**FIGURE 2:**
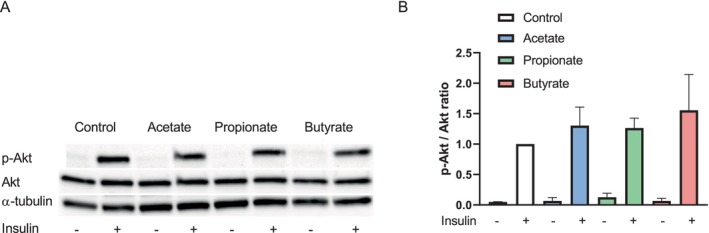
Effect of short‐chain fatty acids (SCFAs) on insulin‐stimulated phosphorylation of Akt. Human myotubes were cultured in 6‐well CellBind culture plates and treated with 100 μM of either acetate, propionate, or butyrate for 24 h before the myotubes were stimulated with or without 100 nM insulin for 15 min. Western Blot (A) analysis of cell lysates using antibodies against phospho‐Akt (P‐Akt, S473), total Akt and α‐tubulin. A representative blot is shown. Densitometry analysis (B) of P‐Akt relative to total Akt. Expression levels were normalised to α‐tubulin and related to the insulin‐treated control. Data are given as mean ± SEM (*n* = 3).

Butyrate has previously been demonstrated to improve insulin sensitivity in C2C12 myoblasts [39]. To examine the effects of SCFAs on insulin signalling in primary human myotubes, we looked at insulin‐stimulated phosphorylation of Akt (Figure 2). Neither of the SCFAs appeared to affect insulin‐stimulated phosphorylation of Akt in the present study (Figure 2A,B).

### 3.3 Effect of SCFAs on Oleic Acid Metabolism

**FIGURE 3:**
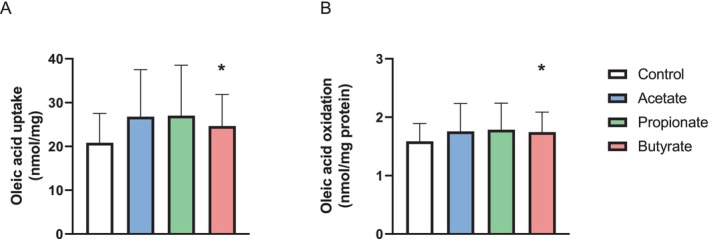
Oleic acid metabolism in human myotubes following short‐chain fatty acid (SCFA) treatment. Human myotubes were cultured in 96‐well CellBind microplates before they were treated with 100 μM of either acetate, propionate, or butyrate for 24 h. The myotubes were then incubated with [1‐^14^C]oleic acid (1 μCi/mL, 100 μM) for 4 h. Oleic acid uptake (A) was assessed as the sum of both oxidised [1‐^14^C]oleic acid and the remaining cell‐associated radioactivity. Oleic acid oxidation (B) refers to the oxidised [1‐^14^C]oleic acid trapped as CO_2_ and counted by liquid scintillation. Data are given as mean ± SEM, *n* = 7 individual experiments with 4 technical replicates per experiment. *p < 0.05 vs. control, paired Student's *t*‐test.

Having investigated the effects of SCFAs on glucose metabolism, we next turned our attention to the putative effect of SCFAs in regulating fatty acid uptake and oxidation in the myotubes (Figure 3). The observed SCFA effects on oleic acid metabolism were modest but with a significant increase in both uptake and oxidation following 100 μM butyrate treatment (Figure 3A,B).

### 3.4 Effects of Palmitate and GLPG0974 on Glucose and Oleic Acid Metabolism

Previous studies have indicated that butyrate can counteract the effect of palmitate [39, 40]. This motivated a comparison of the effects of SCFAs on the uptake and oxidation of glucose and oleic acid in palmitate‐treated human myotubes. Myotubes were treated with 100 μM acetate, propionate, or butyrate in the presence or absence of 300 μM palmitate for 24 h. There were no observable effects of SCFAs on the uptake or oxidation of [^14^C]glucose or [1‐^14^C]oleic acid in the presence of 300 μM palmitate (Figure S3).

GLPG0974 is a known antagonist of the receptor GPR43 and has been shown to inhibit the observed beneficial effects of butyrate in rat aortic endothelial cells [41]. To investigate if glucose and oleic acid metabolism in human myotubes were mediated by GPR43 activation, we treated the cells with 100 μM acetate, propionate, or butyrate in the presence or absence of 100 nM of GLPG0974 for 24 h prior to incubation with either [^14^C]glucose or [^14^C]oleic acid. There were no significant changes in uptake or oxidation of these energy sources in the presence of the GPR43‐antagonist GLPG0974 (Figure S4).

### 3.5 Effects of SCFAs on Protein Synthesis

**FIGURE 4:**
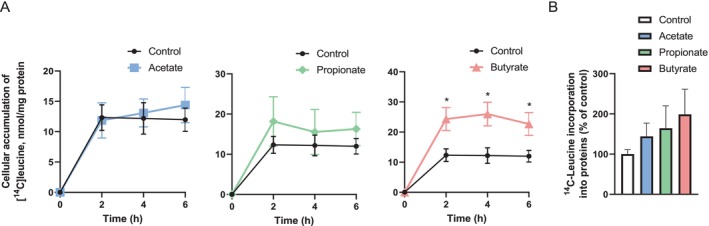
Effect of short‐chain fatty acids (SCFAs) on cell‐associated leucine (A) and incorporation of leucine into cellular proteins (B). (A) Human myoblasts were seeded out in ScintiPlates, grown and differentiated into myotubes. On day 6 of differentiation, the myotubes were treated with 100 μM of either acetate, propionate, or butyrate for 24 h. The myotubes were then incubated with [^14^C]leucine (1 μCi/mL, 0.8 mM) ± their respective SCFA, and the real‐time accumulation of [^14^C]leucine was monitored for 6 h with scintillation proximity assay (SPA) technology. Results are presented as mean ± SEM from four individual experiments (*n* = 4) with 8–16 technical parallels in each experiment. *p < 0.05 versus control, paired Student's *t*‐test. (B) Human myotubes were cultured in 24‐well plates, and on day 6 of differentiation, the myotubes were treated with 100 μM of either acetate or butyrate, or 200 μM propionate, for 24 h together with [^14^C]leucine (1 μCi/mL, 0.8 mM). The cellular proteins were precipitated with trichloroacetic acid and counted by liquid scintillation. Data are given as % of control (100%) mean ± SEM from three individual experiments (*n* = 3) each with 3 technical replicates. The average leucine incorporation in control myotubes was 145 ± 36 nmol/mg cell protein.

As SCFAs have already been established to affect skeletal muscle mass [22], we wanted to investigate whether SCFAs could affect protein synthesis in the myotubes. We assessed cellular accumulation of [^14^C]leucine (Figure 4A) and the incorporation of [^14^C]leucine into proteins (Figure 4B) following myotube treatment with 100 μM of either acetate, propionate, or butyrate. Two different approaches were performed, scintillation proximity assay for [^14^C]leucine accumulation up to 6 h (SPA, Figure 4A) and protein precipitation after [^14^C]leucine incorporation obtained during 24 h incubation (Figure 4B). Interestingly, butyrate induced a rapid leucine accumulation in the myotubes that remained stable for up to 24 h (Figure 4A,B). Although the protein incorporation of leucine after 24 h failed to show a significant difference from the non‐treated control as inferred from Student's *t*‐test, the average level after 24 h corresponded with the established degree of incorporation between 2 and 6 h (Figure 4A). Both experiments indicated about a 2‐fold increase in leucine accumulation and incorporation following butyrate treatment.

### 3.6 Effect of Butyrate on Leucine Incorporation Into Lipids

In view of the subtle effect of butyrate on oleic acid metabolism, as well as the accumulation and incorporation of ^14^C‐leucine into proteins, the incorporation of ^14^C‐leucine into lipids was further analysed. Thin layer chromatography (TLC) was carried out following 24 h treatment with [^14^C]leucine and butyrate (Table 2). Leucine was incorporated into all lipid classes and most in the phospholipid fraction (57%). The total incorporation into lipids (sum of lipids) was not significantly changed by butyrate; however, butyrate reduced the incorporation of leucine in the free fatty acid fraction by more than 50%, indicating that butyrate can affect lipid distribution in myotubes.

**TABLE 2:** Incorporation of [^14^C]leucine (1 μCi/mL, 0.8 mM) into lipids in cultured human myotubes following 24 h of treatment with 100 μM butyrate.

	Phospholipids	Diacylglycerol	Free fatty acids	Triacylglycerol	Cholesterol ester	Sum of lipids
Control	1.60 ± 0.55	0.74 ± 0.06	0.18 ± 0.04	0.25 ± 0.04	0.06 ± 0.01	2.83 ± 0.56
Butyrate	1.62 ± 0.45	0.60 ± 0.15	0.08 ± 0.04*	0.17 ± 0.04	0.06 ± 0.01	2.53 ± 0.58

*Note:* Distribution of lipids in nmol/mg.

* Statistically significant (*p* < 0.05 vs. control, paired Student's *t*‐test), *n* = 3, each with 2 technical parallels.

### 3.7 Acetate and Butyrate as Energy Substrates

**FIGURE 5:**
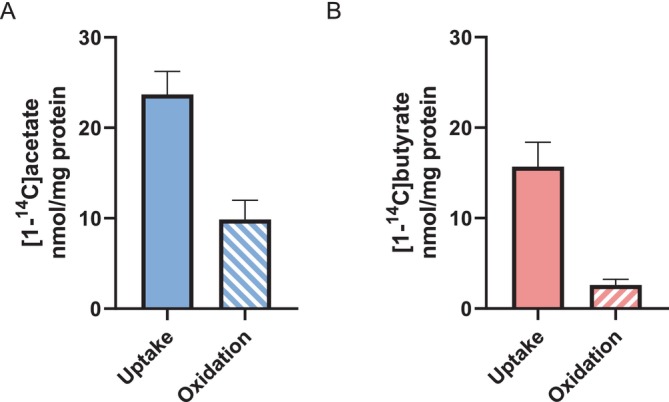
Uptake and oxidation of acetate and butyrate in cultured human myotubes. Human myotubes were cultured in 96‐well CellBind microplates and incubated with (A) [1‐^14^C]acetate (2 μCi/mL, 100 μM) (*n* = 6), or (B) [1‐^14^C]butyrate (0.6 μCi/mL, 12 μM) (*n* = 3) for 4 h after 6–7 days of differentiation. Substrate uptake was assessed as the sum of both oxidised radiolabelled energy substrates and the remaining cell‐associated radioactivity. Oxidation refers to oxidised radiolabelled substrates trapped as CO_2_ in a filter and counted by liquid scintillation. Data are given as mean ^14^C‐labelled substrates (nmol/mg cell protein) ± SEM, *n* = 6 for acetate, 3 for butyrate, and with 4 technical replicates per experiment.

In view of the effect of butyrate on oleic and amino acid metabolism, we investigated the ability of the myotubes to directly oxidise SCFAs. The myotubes were incubated with either [^14^C]acetate or [^14^C]butyrate in the absence of other energy substrates, and uptake and oxidation were measured similarly to glucose and oleic acid (Figures 1 and 3, respectively). Both acetate (Figure 5A) and butyrate (Figure 5B) were taken up by the myotubes, and about 42% of the acetate was oxidised, whereas about 17% of the butyrate was oxidised.

### 3.8 Gene Regulation by SCFAs

**FIGURE 6:**
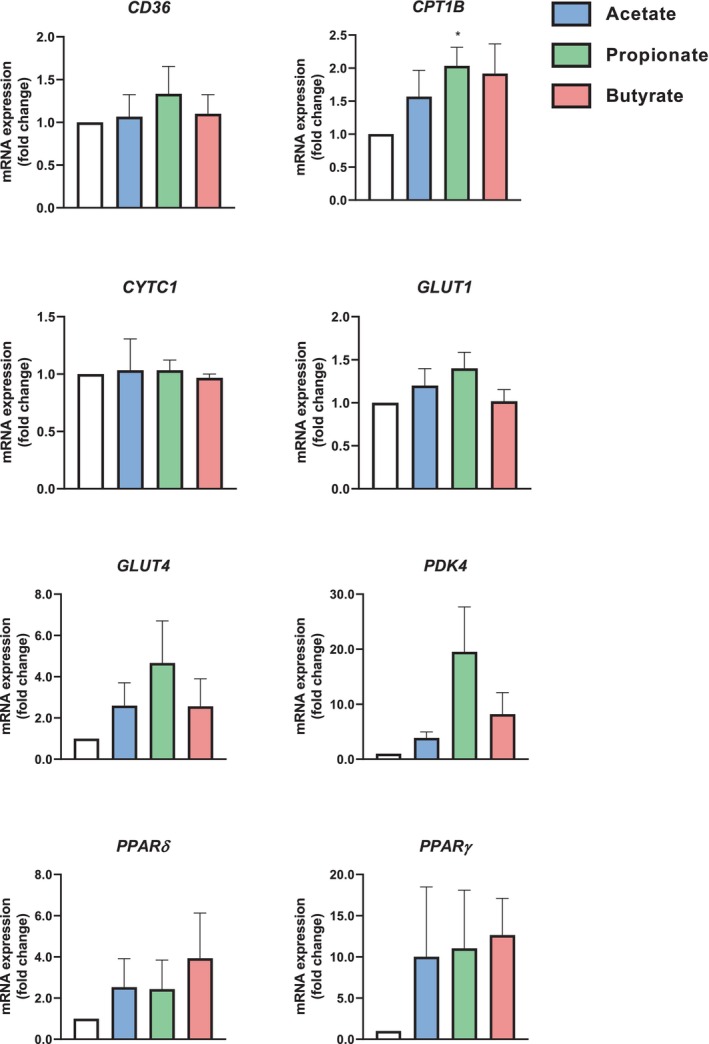
mRNA expression of metabolically relevant genes after myotube treatment with short‐chain fatty acids (SCFAs). Human myotubes were cultured in 6‐well plates, and on day 6 of differentiation, the cells were treated with 100 μM of either acetate, propionate, or butyrate for 24 h. For the assessment of *CYTC1*, 200 μM propionate was used. The mRNA expression was measured by real‐time qPCR and normalised against the housekeeping genes *GAPDH* and/or *RPLP0*. *GLUT1*, *CD36* and *CPT1b* were normalised against either RPLP0 or GAPDH. *CYTC1* was normalised against *RPLP0*, whereas *GLUT4, PDK4, PPARδ* and *PPARγ* were normalised against *GAPDH*. *CD36* = Platelet glycoprotein 4, *CPT1B* = Carnitine palmitoyltransferase 1B, *CYTC1* = Cytochrome C1, *GAPDH* = glyceraldehyde‐3‐phosphate dehydrogenase, *GLUT1* = Solute carrier family 2 facilitated glucose transporter member 1 (*SLC2A1*), *GLUT4* = Solute carrier family 2 facilitated glucose transporter member 4 (*SLC2A4*), *PDK4* = Pyruvate dehydrogenase kinase 4, *PPARδ* = Peroxisome proliferator‐activated receptor δ, *PPARγ* = Peroxisome proliferator‐activated receptor γ, *RPLP0* = Human large ribosomal protein P0. The data are shown as mean ± SEM and the control is set to 1. For *CD36, CPT1B* and *GLUT1*, *n* = 6; for *CYTC1, GLUT4, PDK4, PPARD* and *PPARY*, *n* = 3, with 2 technical replicates of each. * Statistically significant (*p* < 0.05, with one sample *t*‐test).

As shown, SCFAs, particularly butyrate, can regulate energy metabolism. We therefore wanted to examine whether metabolically relevant genes were affected by SCFA treatment. While some of the genes appeared to be regulated by SCFAs, only propionate was observed to increase *CPT1B* mRNA expression (Figure 6).

## 4 Discussion

In this study, we have examined the possible impact of SCFAs on the uptake and utilisation of glucose, oleic acid, and leucine in cultured human myotubes. While there was no observed influence by SCFAs on basal or insulin‐stimulated glucose metabolism, we found that butyrate increased both oleic acid uptake and oxidation, as well as leucine accumulation in human myotubes. We have previously shown that human myotubes express MCTs 1–4 [16], and here we showed that the SCFAs acetate and butyrate are both taken up and utilised as energy sources in cultured primary human myotubes. To the best of our knowledge, this is the first study conducted in primary human myotubes investigating SCFAs ability to modulate energy metabolism and protein synthesis.

We did not observe any marked changes in glucose uptake, nor oxidation or glycogen synthesis following treatment with any of the SCFAs (Figures 1 and 2). In support of these findings, we did not see any significant increase in *GLUT1* or *GLUT4* mRNA expression following SCFA treatment (Figure 6). This was in contrast with previous findings, where insulin‐independent glucose uptake in rat L6 myotubes was increased following 24–48 h acetate treatment [25] and following 30 min propionate treatment in mouse C2C12 myotubes [42]. A combination of all three SCFAs on mouse C2C12 cells has previously been shown to increase glucose uptake [43]; this was however observed following treatment with concentrations much higher than ours (5 mM vs. 100 μM). The conflicting results between our findings and these previous studies may also be due to species differences [44] or variable incubation periods.

We assessed the effect of SCFAs on a selection of metabolically relevant genes, in particular genes involved in fatty acid metabolism and energy selection. While butyrate was shown to slightly increase the uptake and oxidation of oleic acid following 24 h of treatment, this was not accompanied by the increased mRNA expression of the genes expected to be involved in fatty acid metabolism, namely *CD36*, *PDK4*, or *CPT1B* (Figure 6). We did however see a significant increase in *CPT1B* mRNA expression following propionate treatment, but this was not associated with a significantly increased oleic acid oxidation by propionate. However, a larger sample size could potentially provide more sensitivity to detect small changes. A review by Frampton and co‐workers describes that SCFAs are indicated to prevent lipid accumulation in skeletal muscle cells [23], and in line with our findings, SCFAs have previously been shown to increase both the uptake and oxidation of fatty acids [18, 25, 45]. These studies have, however, used either biopsies from rodents or the rat‐derived cell line L6. The myotubes used in this study showed a tendency toward increased mRNA expression of the tested genes following treatment with all SCFAs, which could indicate improved mitochondrial function. These findings were however not significant.

Butyrate has been shown to counteract the inhibitory effect of palmitate on insulin signalling in mouse C2C12 cells [39]. Therefore, we wanted to assess if SCFAs could modify myotube metabolism of glucose and oleic acid in palmitate‐treated human myotubes. As shown in Figure S3, PA increased both glucose and oleic acid uptake and oxidation, and no significant changes were observed in the presence of SCFAs. It can be postulated that the presence of an additional 400 μM non‐labelled fatty acids (100 μM SCFA and 300 μM palmitate) may have masked potential metabolic effects, especially in the case of oleic acid, where the myotubes have taken up and utilised these non‐labelled substrates that are undetectable with the method used.

In previous studies, insulin has been shown to increase glucose uptake by upwards of 50% in human myotubes [46]. Here we show an increase in glucose uptake and oxidation by 22% and 20%, respectively, following treatment with 100 nM insulin (Figure 1). Insulin also induced phosphorylation of Akt and increased glycogen synthesis, here about two‐fold [46]. Treatment with SCFAs alone for 24 h did not affect baseline or insulin‐stimulated phosphorylation of Akt or glycogen synthesis. Previous findings have, however, shown that glycogen content, and thus assumingly glycogen synthesis, was increased in rat muscle 4 h post‐prandially using chow containing acetic acid [47]. Interspecies differences, as well as differences between in vivo and in vitro methodologies, may be the reason for this contrast, but we cannot rule out the possibility of observing a similar effect in human myotubes if they had been treated with SCFAs for a shorter time period. Consistent with the glycogen synthesis results, insulin signalling by phosphorylation of Akt did not appear to be altered following 24 h treatment with the SCFAs (Figure 2). For our experiments, the myotubes used were isolated from lean donors with normal glucose tolerance, so it can be speculated that the results could potentially be more marked by using myotubes isolated from individuals with type 2 diabetes mellitus, or those with obesity. In fact, it has been observed that men with obesity receiving faecal transplants from lean donors had an increase in insulin sensitivity associated with an increase in butyrate producing bacteria in the gut 6 weeks post transplantation [48]. Improved insulin sensitivity in microbiota‐depleted mice receiving faecal transplants from lean, but not obese, mice has previously been described [4]. There have also been observations in murine studies where there was a reduction in body weight and a shift toward improved insulin sensitivity following SCFA treatment in mice with high‐fat diet‐induced obesity [24].

While treatment of the myotubes with SCFAs did not appear to modulate energy metabolism of glucose and oleic acid to a great extent, the SCFAs appeared to increase muscle protein synthesis, as shown by SPA (Figure 4A). Although all three of the SCFAs seemed to increase the accumulation and incorporation of leucine into cellular protein, butyrate had the most profound effect and was the only one showing a statistical significance. We also found that butyrate treatment reduced incorporation of leucine‐derived carbons into free fatty acids, while the total lipid content remained the same. Interestingly, most of the data available regarding the effects of SCFAs on protein synthesis are with butyrate. Butyrate has previously been shown to exert growth‐promoting properties in an intestinal porcine epithelial cell model (IPEC‐J2), which was suggested to be through activation of the Akt/mTOR mediated protein synthesis pathway [49]. In contrast to our study, these epithelial cells were cultured in 1 mM butyrate‐containing media for the entire differentiation period, and the Akt/mTOR pathway was activated by lipopolysaccharide [49]. Although our findings did not reveal phosphorylation of Akt following 24 h of SCFA treatment, we cannot rule out that a different treatment duration could activate Akt. Alternatively, butyrate might activate other protein synthesis‐promoting pathways or, suggestively, act similarly to the chemically related structure β‐hydroxy‐β‐methyl butyrate (HMB), which was previously found to suppress protein degradation in C2C12 myotubes [50]. Several studies have found butyrate to have protective effects on muscle atrophy, both when measuring levels of butyrate in serum from human subjects [51, 52], and in muscle tissue from mice [21]. However, it has been suggested that the SCFAs might not influence skeletal muscle mass in conditions where there is an energy surplus, such as obesity, but are rather beneficial when there is an increased metabolic demand, for instance during developmental growth, fasting, exercise and ageing [23]. In support of this, one study found that butyrate contributes to postnatal muscle growth in piglets through enhanced satellite cell myogenesis, increased myonuclear accretion and subsequent myofiber hypertrophy [53].

SCFAs may contribute to myotube metabolism by several mechanisms. SCFAs have been established to be ligands of the membrane bound G protein‐coupled receptors GPR41 and GPR43 [26, 27, 28]. The receptors' role in muscle metabolism is however still poorly understood, and there remains a need for further studies. Neither glucose nor oleic acid metabolism was altered using the GPR43 antagonist GLPG0974 in the present study.

It can be argued that limitations of the experiments include the concentrations of the individual SCFAs, as well as the experimental durations. The concentrations of the SCFAs in the current study were above the physiological concentrations. Additionally, in a physiological milieu, the SCFAs may potentially function in tandem, as well as more acutely than 24 h. In this study, the SCFA concentrations used have ranged from 10 to 1000 μM, which for at least propionate and butyrate surpasses physiologically observed concentrations [7, 8]. In IPEC‐J2 cells, no significantly adverse effects were observed using 1 mM butyrate [49]. The 100–200 μM of SCFAs used in this study is closer to the physiological range than many other in vitro studies (0.5–5 mM) [25, 43, 49, 54]. This may account for some of the discrepancies between our findings and other studies. We did, as shown in Figure S1, not see any differences in glucose or oleic acid metabolism when increasing the SCFA concentrations. Still, while systemic concentrations of the SCFAs may remain relatively low, they may still play a direct role as both regulators and energy sources in human skeletal muscle, or they may affect the myotubes indirectly by their use in secondary metabolite production [23].

The findings of the current study suggest that SCFAs, especially butyrate, may potentially have clinical applications. Butyrate was shown to increase both uptake and oxidation of oleic acid, indicating a beneficial role in metabolic health. Additionally, our findings indicate that butyrate may have some anabolic properties in human skeletal muscle, which might be relevant. Still, further studies are needed to examine the possible therapeutic potential of SCFAs.

In conclusion, we have shown that SCFAs elicit metabolic effects on cultured human myotubes. The separate SCFAs can be metabolised to different extents, and they exert different roles in modifying leucine accumulation as well as incorporation into free fatty acids. In the current study, butyrate emerges as the most effective SCFA in regulating both oleic acid and leucine metabolisms in cultured human skeletal muscle cells.

## Author Contributions

Conceptualisation: R.H.T., V.A., O.W., A.C.R., V.H.T.‐H., M.C.W.M. Funding: V.A., M.C.W.M., V.H.T.‐H. (Oslo Metropolitan University). Methodology: R.H.T., C.S., N.G.L., V.A., O.W., S.B., S.H.S. Manuscript preparation: R.H.T., V.A., O.W. Visualisation: R.H.T. Project administration: R.H.T. Formal analysis: R.H.T., V.A., O.W., A.C.R., L.E. Data curation: R.H.T., O.W., V.A. Review and editing: R.H.T., V.A., O.W., V.H.T.‐H., M.C.W.M., L.E., A.C.R. All authors have read and agreed to the published version of the manuscript.

## Ethics Statement

Human skeletal muscle biopsies were obtained after informed patient written consent and ethical approval by the Regional Committee for Medical and Health Research Ethics South‐East, Oslo, Norway (reference number: REK11959) or from the West of Scotland Research Ethics Committee 4 (reference number: 16/WS/0002). The study was conducted in accordance with the guidelines of the Declaration of Helsinki.

## Conflicts of Interest

The authors declare no conflicts of interest.

## Supporting information


**Figure S1.** Time‐dependent effects of SCFAs on glucose and oleic acid metabolism.
**Figure S2.** Concentration‐dependent effects of SCFAs on glucose and oleic acid metabolism.
**Figure S3.** Effect of SCFA treatment in combination with palmitate on glucose and oleic acid metabolism in human myotubes.
**Figure S4.** Effects of SCFA treatments in combination with the GPR43 antagonist GLPG0974 on glucose and oleic acid metabolism in human myotubes.

## Data Availability

The data that support the findings of this study are available from the corresponding author upon reasonable request.

## References

[1] E. E. Blaak , E. E. Canfora , S. Theis , et al., “Short Chain Fatty Acids in Human Gut and Metabolic Health,” Beneficial Microbes 11, no. 5 (2020): 411–455, 10.3920/BM2020.0057. 32865024

[2] A. M. Stephen , M. M. J. Champ , S. J. Cloran , et al., “Dietary Fibre in Europe: Current State of Knowledge on Definitions, Sources, Recommendations, Intakes and Relationships to Health,” Nutrition Research Reviews 30 (2017): 149–190, 10.1017/S095442241700004X. 28676135

[3] E. E. Canfora , R. C. R. Meex , K. Venema , and E. E. Blaak , “Gut Microbial Metabolites in Obesity, NAFLD and T2DM,” Nature Reviews. Endocrinology 15 (2019): 261–273, 10.1038/s41574-019-0156-z. 30670819

[4] Z. Li , E. Zhou , C. Liu , et al., “Dietary Butyrate Ameliorates Metabolic Health Associated With Selective Proliferation of Gut Lachnospiraceae Bacterium 28‐4,” JCI Insight 8 (2023): e166655, 10.1172/jci.insight.166655. 36810253 PMC9977501

[5] A. Schwiertz , D. Taras , K. Schäfer , et al., “Microbiota and SCFA in Lean and Overweight Healthy Subjects,” Obesity (Silver Spring) 18 (2010): 190–195, 10.1038/oby.2009.167. 19498350

[6] S. Kim , J. H. Kim , B. O. Park , and Y. S. Kwak , “Perspectives on the Therapeutic Potential of Short‐Chain Fatty Acid Receptors,” BMB Reports 47 (2014): 173–178, 10.5483/bmbrep.2014.47.3.272. 24499669 PMC4163876

[7] J. G. Bloemen , K. Venema , M. C. van de Poll , S. W. Olde Damink , W. A. Buurman , and C. H. Dejong , “Short Chain Fatty Acids Exchange Across the Gut and Liver in Humans Measured at Surgery,” Clinical Nutrition 28 (2009): 657–661, 10.1016/j.clnu.2009.05.011. 19523724

[8] J. G. Bloemen , S. W. M. Olde Damink , K. Venema , W. A. Buurman , R. Jalan , and C. H. C. Dejong , “Short Chain Fatty Acids Exchange: Is the Cirrhotic, Dysfunctional Liver Still Able to Clear Them?,” Clinical Nutrition 29 (2010): 365–369, 10.1016/j.clnu.2009.10.002. 19897285

[9] C. J. Kelly , L. Zheng , E. L. Campbell , et al., “Crosstalk Between Microbiota‐Derived Short‐Chain Fatty Acids and Intestinal Epithelial HIF Augments Tissue Barrier Function,” Cell Host & Microbe 17 (2015): 662–671, 10.1016/j.chom.2015.03.005. 25865369 PMC4433427

[10] H. B. Wang , P. Y. Wang , X. Wang , Y. L. Wan , and Y. C. Liu , “Butyrate Enhances Intestinal Epithelial Barrier Function via Up‐Regulation of Tight Junction Protein Claudin‐1 Transcription,” Digestive Diseases and Sciences 57 (2012): 3126–3135, 10.1007/s10620-012-2259-4. 22684624

[11] E. S. Chambers , T. Preston , G. Frost , and D. J. Morrison , “Role of Gut Microbiota‐Generated Short‐Chain Fatty Acids in Metabolic and Cardiovascular Health,” Current Nutrition Reports 7 (2018): 198–206, 10.1007/s13668-018-0248-8. 30264354 PMC6244749

[12] R. Mirzaei , A. Afaghi , S. Babakhani , et al., “Role of Microbiota‐Derived Short‐Chain Fatty Acids in Cancer Development and Prevention,” Biomedicine & Pharmacotherapy 139 (2021): 111619, 10.1016/j.biopha.2021.111619. 33906079

[13] K. A. Krautkramer , J. Fan , and F. Backhed , “Gut Microbial Metabolites as Multi‐Kingdom Intermediates,” Nature Reviews. Microbiology 19 (2021): 77–94, 10.1038/s41579-020-0438-4. 32968241

[14] A. Ritzhaupt , I. S. Wood , A. Ellis , K. B. Hosie , and S. P. Shirazi‐Beechey , “Identification and Characterization of a Monocarboxylate Transporter (MCT1) in Pig and Human Colon: Its Potential to Transport L‐Lactate as Well as Butyrate,” Journal of Physiology 513, no. Pt 3 (1998): 719–732, 10.1111/j.1469-7793.1998.719ba.x. 9824713 PMC2231331

[15] I. Moschen , A. Broer , S. Galic , F. Lang , and S. Broer , “Significance of Short Chain Fatty Acid Transport by Members of the Monocarboxylate Transporter Family (MCT),” Neurochemical Research 37 (2012): 2562–2568, 10.1007/s11064-012-0857-3. 22878645

[16] J. Lund , V. Aas , R. H. Tingstad , A. Van Hees , and N. Nikolic , “Utilization of Lactic Acid in Human Myotubes and Interplay With Glucose and Fatty Acid Metabolism,” Scientific Reports 8 (2018): 9814, 10.1038/s41598-018-28249-5. 29959350 PMC6026123

[17] R. A. Carey and D. Montag , “Exploring the Relationship Between Gut Microbiota and Exercise: Short‐Chain Fatty Acids and Their Role in Metabolism,” BMJ Open Sport & Exercise Medicine 7 (2021): e000930, 10.1136/bmjsem-2020-000930. PMC8061837 33981447

[18] Z. Gao , J. Yin , J. Zhang , et al., “Butyrate Improves Insulin Sensitivity and Increases Energy Expenditure in Mice,” Diabetes 58 (2009): 1509–1517, 10.2337/db08-1637. 19366864 PMC2699871

[19] T. M. Henagan , B. Stefanska , Z. Fang , et al., “Sodium Butyrate Epigenetically Modulates High‐Fat Diet‐Induced Skeletal Muscle Mitochondrial Adaptation, Obesity and Insulin Resistance Through Nucleosome Positioning,” British Journal of Pharmacology 172 (2015): 2782–2798, 10.1111/bph.13058. 25559882 PMC4439875

[20] J. H. Pan , J. H. Kim , H. M. Kim , et al., “Acetic Acid Enhances Endurance Capacity of Exercise‐Trained Mice by Increasing Skeletal Muscle Oxidative Properties,” Bioscience, Biotechnology, and Biochemistry 79 (2015): 1535–1541, 10.1080/09168451.2015.1034652. 26000971

[21] M. E. Walsh , A. Bhattacharya , K. Sataranatarajan , et al., “The Histone Deacetylase Inhibitor Butyrate Improves Metabolism and Reduces Muscle Atrophy During Aging,” Aging Cell 14 (2015): 957–970, 10.1111/acel.12387. 26290460 PMC4693467

[22] M. S. Lustgarten , “The Role of the Gut Microbiome on Skeletal Muscle Mass and Physical Function: 2019 Update,” Frontiers in Physiology 10 (2019): 1435, 10.3389/fphys.2019.01435. 31911785 PMC6933299

[23] J. Frampton , K. G. Murphy , G. Frost , and E. S. Chambers , “Short‐Chain Fatty Acids as Potential Regulators of Skeletal Muscle Metabolism and Function,” Nature Metabolism 2 (2020): 840–848, 10.1038/s42255-020-0188-7. 32694821

[24] G. den Besten , A. Bleeker , A. Gerding , et al., “Short‐Chain Fatty Acids Protect Against High‐Fat Diet–Induced Obesity via a PPARγ‐Dependent Switch From Lipogenesis to Fat Oxidation,” Diabetes 64, no. 7 (2015): 2398–2408, 10.2337/db14-1213. 25695945

[25] H. Maruta , Y. Yoshimura , A. Araki , M. Kimoto , Y. Takahashi , and H. Yamashita , “Activation of AMP‐Activated Protein Kinase and Stimulation of Energy Metabolism by Acetic Acid in L6 Myotube Cells,” PLoS One 11 (2016): e0158055, 10.1371/journal.pone.0158055. 27348124 PMC4922563

[26] A. J. Brown , S. M. Goldsworthy , A. A. Barnes , et al., “The Orphan G Protein‐Coupled Receptors GPR41 and GPR43 Are Activated by Propionate and Other Short Chain Carboxylic Acids,” Journal of Biological Chemistry 278 (2003): 11312–11319, 10.1074/jbc.M211609200. 12496283

[27] N. E. Nilsson , K. Kotarsky , C. Owman , and B. Olde , “Identification of a Free Fatty Acid Receptor, FFA2R, Expressed on Leukocytes and Activated by Short‐Chain Fatty Acids,” Biochemical and Biophysical Research Communications 303 (2003): 1047–1052, 10.1016/s0006-291x(03)00488-1. 12684041

[28] E. E. Canfora , J. W. Jocken , and E. E. Blaak , “Short‐Chain Fatty Acids in Control of Body Weight and Insulin Sensitivity,” Nature Reviews. Endocrinology 11 (2015): 577–591, 10.1038/nrendo.2015.128. 26260141

[29] J. A. Bonini , S. M. Anderson , and D. F. Steiner , “Molecular Cloning and Tissue Expression of a Novel Orphan G Protein‐Coupled Receptor From Rat Lung,” Biochemical and Biophysical Research Communications 234 (1997): 190–193, 10.1006/bbrc.1997.6591. 9168987

[30] A. S. Husted , M. Trauelsen , O. Rudenko , S. A. Hjorth , and T. W. Schwartz , “GPCR‐Mediated Signaling of Metabolites,” Cell Metabolism 25 (2017): 777–796, 10.1016/j.cmet.2017.03.008. 28380372

[31] M. Bellahcene , J. F. O'Dowd , E. T. Wargent , et al., “Male Mice That Lack the G‐Protein‐Coupled Receptor GPR41 Have Low Energy Expenditure and Increased Body Fat Content,” British Journal of Nutrition 109 (2013): 1755–1764, 10.1017/S0007114512003923. 23110765

[32] I. Kimura , K. Ozawa , D. Inoue , et al., “The Gut Microbiota Suppresses Insulin‐Mediated Fat Accumulation via the Short‐Chain Fatty Acid Receptor GPR43,” Nature Communications 4 (2013): 1829, 10.1038/ncomms2852. PMC3674247 23652017

[33] M. Gaster , S. R. Kristensen , H. Beck‐Nielsen , and H. D. Schroder , “A Cellular Model System of Differentiated Human Myotubes,” APMIS 109 (2001): 735–744. 11900052 10.1034/j.1600-0463.2001.d01-140.x

[34] R. H. Tingstad , F. Norheim , F. Haugen , et al., “The Effect of Toll‐Like Receptor Ligands on Energy Metabolism and Myokine Expression and Secretion in Cultured Human Skeletal Muscle Cells,” Scientific Reports 11 (2021): 24219, 10.1038/s41598-021-03730-w. 34930972 PMC8688447

[35] A. J. Wensaas , A. C. Rustan , K. Lövstedt , et al., “Cell‐Based Multiwell Assays for the Detection of Substrate Accumulation and Oxidation,” Journal of Lipid Research 48 (2007): 961–967. 17213484 10.1194/jlr.D600047-JLR200

[36] M. M. Bradford , “A Rapid and Sensitive Method for the Quantitation of Microgram Quantities of Protein Utilizing the Principle of Protein‐Dye Binding,” Analytical Biochemistry 72 (1976): 248–254, 10.1006/abio.1976.9999. 942051

[37] J. Franch , R. Aslesen , and J. Jensen , “Regulation of Glycogen Synthesis in Rat Skeletal Muscle After Glycogen‐Depleting Contractile Activity: Effects of Adrenaline on Glycogen Synthesis and Activation of Glycogen Synthase and Glycogen Phosphorylase,” Biochemical Journal 344, no. Pt 1 (1999): 231–235. 10548555 PMC1220635

[38] M. Gaster , A. C. Rustan , V. Aas , and H. Beck‐Nielsen , “Reduced Lipid Oxidation in Skeletal Muscle From Type 2 Diabetic Subjects May Be of Genetic Origin: Evidence From Cultured Myotubes,” Diabetes 53 (2004): 542–548. 14988236 10.2337/diabetes.53.3.542

[39] M. Rios‐Morales , M. A. Vieira‐Lara , E. Homan , et al., “Butyrate Oxidation Attenuates the Butyrate‐Induced Improvement of Insulin Sensitivity in Myotubes,” Biochimica et Biophysica Acta – Molecular Basis of Disease 1868 (2022): 166476, 10.1016/j.bbadis.2022.166476. 35811030

[40] P. P. Hommelberg , P. P. H. Hommelberg , J. Plat , et al., “Palmitate‐Induced Skeletal Muscle Insulin Resistance Does Not Require NF‐κB Activation,” Cellular and Molecular Life Sciences 68, no. 7 (2010): 1215–1225, 10.1007/s00018-010-0515-3. 20820848 PMC3056136

[41] I. Robles‐Vera , M. Toral , N. de la Visitación , N. Aguilera‐Sánchez , J. M. Redondo , and J. Duarte , “Protective Effects of Short‐Chain Fatty Acids on Endothelial Dysfunction Induced by Angiotensin II,” Frontiers in Physiology 11 (2020): 277, 10.3389/fphys.2020.00277. 32372967 PMC7176911

[42] J. H. Han , I. S. Kim , S. H. Jung , S. G. Lee , H. Y. Son , and C. S. Myung , “The Effects of Propionate and Valerate on Insulin Responsiveness for Glucose Uptake in 3T3‐L1 Adipocytes and C2C12 Myotubes via G Protein‐Coupled Receptor 41,” PLoS One 9 (2014): e95268, 10.1371/journal.pone.0095268. 24748202 PMC3991595

[43] B. M. J. Otten , M. Sthijns , and F. J. Troost , “A Combination of Acetate, Propionate, and Butyrate Increases Glucose Uptake in C2C12 Myotubes,” Nutrients 15, no. 4 (2023): 946, 10.3390/nu15040946. 36839304 PMC9967986

[44] A. M. Abdelmoez , L. Sardón Puig , J. A. B. Smith , et al., “Comparative Profiling of Skeletal Muscle Models Reveals Heterogeneity of Transcriptome and Metabolism,” American Journal of Physiology. Cell Physiology 318 (2020): C615–C626, 10.1152/ajpcell.00540.2019. 31825657 PMC7099524

[45] J. Hong , Y. Jia , S. Pan , et al., “Butyrate Alleviates High Fat Diet‐Induced Obesity Through Activation of Adiponectin‐Mediated Pathway and Stimulation of Mitochondrial Function in the Skeletal Muscle of Mice,” Oncotarget 7 (2016): 56071–56082, 10.18632/oncotarget.11267. 27528227 PMC5302897

[46] V. Aas , S. S. Bakke , Y. Z. Feng , et al., “Are Cultured Human Myotubes Far From Home?,” Cell and Tissue Research 354 (2013): 671–682, 10.1007/s00441-013-1655-1. 23749200

[47] T. Fushimi and Y. Sato , “Effect of Acetic Acid Feeding on the Circadian Changes in Glycogen and Metabolites of Glucose and Lipid in Liver and Skeletal Muscle of Rats,” British Journal of Nutrition 94 (2005): 714–719, 10.1079/bjn20051545. 16277773

[48] A. Vrieze , E. van Nood , F. Holleman , et al., “Transfer of Intestinal Microbiota From Lean Donors Increases Insulin Sensitivity in Individuals With Metabolic Syndrome,” Gastroenterology 143 (2012): 913–916.e917, 10.1053/j.gastro.2012.06.031. 22728514

[49] H. Yan and K. M. Ajuwon , “Butyrate Modifies Intestinal Barrier Function in IPEC‐J2 Cells Through a Selective Upregulation of Tight Junction Proteins and Activation of the Akt Signaling Pathway,” PLoS One 12 (2017): e0179586, 10.1371/journal.pone.0179586. 28654658 PMC5487041

[50] Y. Duan , Y. Zhong , B. Song , et al., “Suppression of Protein Degradation by Leucine Requires Its Conversion to β‐Hydroxy‐β‐Methyl Butyrate in C2C12 Myotubes,” Aging (Albany NY) 11, no. 24 (2019): 11922–11936, 10.18632/aging.102509. 31881014 PMC6949090

[51] W. Q. Lv , X. Lin , H. Shen , et al., “Human Gut Microbiome Impacts Skeletal Muscle Mass via Gut Microbial Synthesis of the Short‐Chain Fatty Acid Butyrate Among Healthy Menopausal Women,” Journal of Cachexia, Sarcopenia and Muscle 12 (2021): 1860–1870, 10.1002/jcsm.12788. 34472211 PMC8718076

[52] G. Tang , Y. du , H. Guan , et al., “Butyrate Ameliorates Skeletal Muscle Atrophy in Diabetic Nephropathy by Enhancing Gut Barrier Function and FFA2‐Mediated PI3K/Akt/mTOR Signals,” British Journal of Pharmacology 179 (2022): 159–178, 10.1111/bph.15693. 34638162

[53] R. L. Murray , W. Zhang , M. Iwaniuk , E. Grilli , and C. H. Stahl , “Dietary Tributyrin, an HDAC Inhibitor, Promotes Muscle Growth Through Enhanced Terminal Differentiation of Satellite Cells,” Physiological Reports 6 (2018): e13706, 10.14814/phy2.13706. 29845774 PMC5974723

[54] K. Van , J. L. Burns , and J. M. Monk , “Effect of Short‐Chain Fatty Acids on Inflammatory and Metabolic Function in an Obese Skeletal Muscle Cell Culture Model,” Nutrients 16, no. 4 (2024): 500, 10.3390/nu16040500. 38398822 PMC10891728

