# Supplementary material for: Impact of Short‐Chain Fatty Acids on Glucose, Fatty Acid and Leucine Metabolism in Primary Human Myotubes
Source: Endocrinol Diabetes Metab. 2025 Mar 7;8(2):e70042. doi: 10.1002/edm2.70042 (PMC11885951; doi:10.1002/edm2.70042)
Supplement: Supplementary file 1 — Figure S1. Time‐dependent effects of SCFAs on glucose and oleic acid metabolism. Figure S2. Concentration‐dependent effects of SCFAs on glucose and oleic acid metabolism. Figure S3. Effect of SCFA treatment in combination with palmitate on glucose and oleic acid metabolism in human myotubes. Figure S4. Effects of SCFA treatments in combination with the GPR43 antagonist GLPG0974 on glucose and oleic acid metabolism in human myotubes. [file EDM2-8-e70042-s001.docx]

**Supplementary figure 1: Time dependent effects of SCFAs on glucose and oleic acid metabolism**

Supplementary figure 1: Glucose and oleic acid metabolism in cultured human myotubes following short-chain fatty acid (SCFA) treatment at different durations. Human myotubes were cultured in 96-well CellBind microplates before they were treated with either 100 µM acetate (A, D), 200 µM propionate (B, D) or 100 µM butyrate (C, D) for 24 h, 48 h or 96 h prior to uptake and oxidation assay. For the uptake and oxidation assays, the cells were then incubated with either D-[^14^C(U)]glucose (1 µCi/mL, 200 µM) or [1-^14^C]oleic acid (1 µCi/mL, 100 µM) for 4 h. Energy uptake was assessed as the sum of both oxidized D-[^14^C(U)]glucose or [1-^14^C]oleic acid and the remaining cell associated radioactivity. Oxidation refers to the oxidized D-[^14^C(U)]glucose or
[1-^14^C]oleic acid trapped as CO_2_ and counted by liquid scintillation. Data are given as nmol/mg of cell protein, n = 6 experiments with 4 technical replicates per experiment.

**Supplementary figure 2: Concentration-dependent effects of SCFAs on glucose and oleic acid metabolism**

Supplementary figure 2: Glucose and oleic acid metabolism in cultured human myotubes following treatment with short-chain fatty acids (SCFAs) at different concentrations. Human myotubes were cultured in 96-well CellBind microplates before they were treated with 100, 500 or 1000 µM of acetate (A, D), 10, 100 or 200 µM of propionate (B, E) or 10, 100 or 200 µM butyrate (C, F) for 24 h. For the uptake and oxidation assays, the cells were then incubated with either D-[^14^C(U)]glucose (1 µCi/mL, 200 µM) or [1-^14^C]oleic acid (1 µCi/mL, 100 µM) for 4 h. Substrate uptake was assessed as the sum of both oxidized D-[^14^C(U)]glucose or [1-^14^C]oleic acid and the remaining cell associated radioactivity. Oxidation refers to oxidized D-[^14^C(U)]glucose or [1-^14^C]oleic acid trapped as CO_2_ and counted by liquid scintillation. Data are given as % of control mean with SEM and the average glucose uptake and oxidation in untreated control cells were 6.5 ± 1.1 and 1.3 ± 0.4 nmol/mg cell protein, respectively, and the average oleic acid uptake and oxidation in untreated control cells were 32.0 ± 7.8 and 1.7 ± 0.5 nmol/mg cell protein, respectively. n = 4 experiments with 4 technical replicates per experiment.

**Supplementary figure 3: Effect of SCFA treatment in combination with palmitate on glucose and oleic acid metabolism in human myotubes**

Supplementary figure 3: Glucose and oleic acid metabolism in cultured human myotubes following short-chain fatty acid (SCFA) treatment in the presence or absence of palmitate (300 µM). Human myotubes were cultured in 96-well CellBind microplates before they were treated with 100 µM of either acetate, propionate or butyrate in the presence or absence of 300 µM palmitate for 24 h. For the uptake and oxidation assays, the cells were then incubated with either D-[^14^C(U)]glucose (1 µCi/mL, 200 µM) or [1-^14^C]oleic acid (1 µCi/mL, 100 µM) for 4 h. Glucose uptake [A] was assessed as the sum of both oxidized D-[^14^C(U)]glucose and the remaining cell associated radioactivity. Glucose oxidation [B] refers to oxidized D-[^14^C(U)]glucose trapped as CO_2_ and counted by liquid scintillation. Oleic acid uptake [C] and oleic acid oxidation [D] was assessed as described for glucose. Data are given as % of mean of SCFA treatment only with SEM, and the average glucose uptake and oxidation in untreated control cells were 625.1 ± 193.0 and 542.4 ± 183.3 nmol/mg cell protein, respectively. For oleic acid, the average uptake and oxidation in untreated control cells were 5.9 ± 0.3 and 1.3 ± 0.1 nmol/mg cell protein, respectively. n = 3 experiments with 4 technical replicates per experiment.

**Supplementary figure 4: Effects of SCFA treatments in combination with the GPR43 antagonist GLPG0974** **on glucose and oleic acid metabolism in human myotubes**

Supplementary figure 4: Glucose and oleic acid metabolism in cultured human myotubes following short-chain fatty acid (SCFA) treatment in the presence or absence of the GPR43 antagonist GLPG0974. Human myotubes were cultured in 96-well CellBind microplates before they were treated with 100 µM of either acetate, propionate or butyrate in the presence or absence of 100 nM GLPG0974 for 24 h. For the uptake and oxidation assays, the cells were then incubated with either D-[^14^C(U)]glucose (1 µCi/mL, 200 µM) or [1-^14^C]oleic acid (1 µCi/mL, 100 µM) for 4 h. Glucose uptake [A] was assessed as the sum of both oxidized D-[^14^C(U)]glucose and the remaining cell associated radioactivity. Glucose oxidation [B] refers to the oxidized D-[^14^C(U)]glucose trapped in a filter as CO_2_ and counted by liquid scintillation. Oleic acid uptake [C] was assessed as the sum of both oxidized [1-^14^C]oleic acid and the remaining cell associated radioactivity. Oleic acid oxidation [D] refers to the oxidized [1-^14^C]oleic acid trapped as CO_2_ and counted by liquid scintillation. Data are given as % of control mean with SEM and the average glucose uptake and oxidation in untreated control cells were 625.1 ± 193.0 and 542.4 ± 183.3 nmol/mg cell protein, respectively, and the average oleic acid uptake and oxidation in untreated control cells were 5.9 ± 0.3 and 1.3 ± 0.1 nmol/mg cell protein, respectively. n= 3-10 experiments with 4 technical replicates per experiment.
